# Dural Puncture Epidural, Standard Epidural, and No Epidural: Comparative Effects on Labor Progression and Neonatal Outcomes

**DOI:** 10.3390/jcm15166380

**Published:** 2026-08-18

**Authors:** Kubra C. Yilmaz, Humeyra E. Ates Eminoglu, Mariah Arif, Tugba Aksungur, Gul C. Colak, Seyhmus Tunc, Mehmet B. Tozoglu, Ahmet S. Saracoglu, Fatma Acil, Mustafa Bicak, Kevser Arkan

**Affiliations:** 1 Department of Obstetrics and Gynecology, Gazi Yaşargil Training and Research Hospital, 21500 Diyarbakır, Turkey; dr.humeyraates@gmail.com (H.E.A.E.); tugbaaksngr@gmail.com (T.A.); cvs_gul_21@hotmail.com (G.C.C.); drseyhmustunc@hotmail.com (S.T.); 2 Department of Anesthesiology, ICU, and Perioperative Medicine, Hamad General Hospital, Hamad Medical Corporation, Doha P.O. Box 3050, Qatar; 3 College of Liberal Arts and Sciences, University of Florida, Gainesville, FL 32611, USA; mehmettozoglub@gmail.com; 4 Paxon School for Advanced Studies, Jacksonville, FL 32254, USA; sarpjax@gmail.com; 5 Department of Anesthesiology, Gazi Yaşargil Training and Research Hospital, 21070 Diyarbakır, Turkey; acilfatma@gmail.com (F.A.); drmustafabicak@gmail.com (M.B.); 6 Department of Obstetrics and Gynecology, Optimed Private Hospital, 59500 Tekirdag, Turkey; kevser.toprak1989@gmail.com

**Keywords:** dural puncture epidural analgesia, epidural analgesia, labor analgesia, second stage of labor, umbilical cord pH, neonatal outcomes

## Abstract

**Objective:** To compare non-epidural labor, standard epidural analgesia, and dural puncture epidural analgesia with respect to labor progression, obstetric outcomes, analgesia-related outcomes, and neonatal results. **Methods:** This single-center retrospective observational cohort study included 168 term singleton parturients: 58 without epidural analgesia, 56 with standard epidural analgesia, and 54 with dural puncture epidural analgesia. The primary outcome was the duration of the second stage of labor. Secondary outcomes included first-stage duration, mode of delivery, blood loss, transfusion, analgesia quality, maternal side effects, umbilical venous pH, biochemical acidosis, Apgar scores, neonatal intensive care unit admission, and neonatal resuscitation. Analyses included group comparisons, adjusted models, quantile regression, Firth penalized logistic regression, propensity-score inverse probability weighting, and a nulliparous sensitivity analysis. **Results:** Second-stage duration was comparable across groups (median 40.0, 45.0, and 48.5 min in the no-epidural, standard epidural, and dural puncture epidural groups, respectively; *p* = 0.118). Active pushing time was likewise comparable (8.0, 8.5, and 12.0 min, respectively; *p* = 0.292), and quantile regression, together with a nulliparous sensitivity analysis—in which the second stage was longest in the no-epidural group—did not indicate epidural-related labor prolongation. Compared with standard epidural analgesia, dural puncture epidural analgesia showed numerically lower pain scores and a numerically lower rescue bolus requirement (30/54 [55.6%] vs. 39/56 [69.6%]; *p* = 0.183), although neither difference reached statistical significance. Umbilical cord venous pH and biochemical acidosis (cord venous pH < 7.20) did not differ significantly among groups (*p* = 0.266 and *p* = 0.335, respectively). The only neonatal endpoint that differed between groups was a recorded fetal acidosis flag, which was more frequent in the epidural groups (0%, 7.1%, and 16.7%; *p* = 0.004) but was not corroborated by objective cord pH, Apgar scores, neonatal resuscitation, or a significant increase in neonatal intensive care unit admission (5.2%, 10.7%, and 16.7%; *p* = 0.145). **Conclusions:** Contemporary low-dose standard epidural and dural puncture epidural analgesia were not associated with clinically meaningful prolongation of labor, active pushing time, or major obstetric intervention. Dural puncture epidural analgesia showed a non-significant trend toward more favorable analgesic performance compared with standard epidural analgesia. Objective neonatal acid–base measures (cord venous pH and biochemical acidosis) did not differ significantly between groups; only a recorded fetal acidosis flag was more frequent in the epidural groups, without correlation to objective cord pH or to adverse clinical neonatal outcomes. These results support the obstetric and neonatal safety of contemporary low-dose epidural techniques; the isolated recorded acidosis signal warrants confirmation in prospective studies with standardized cord blood gas sampling.

## 1. Introduction

Neuraxial analgesia remains the most effective method for pain relief during labor and is a central component of contemporary obstetric anesthesia practice. Although epidural analgesia has historically been associated with concerns regarding prolonged labor, increased operative delivery, and adverse neonatal outcomes, the interpretation of these associations has evolved substantially with the use of low-concentration local anesthetic-opioid regimens, patient-controlled epidural analgesia, and programmed intermittent epidural bolus techniques. Recent reviews emphasize that many previously reported adverse associations are likely influenced by confounding by indication, differences in obstetric management, and the limitation of observational study designs rather than by a direct causal effect of epidural analgesia itself [1,2]. Building on this premise, we hypothesized a priori that contemporary low-dose epidural techniques would not prolong labor or increase obstetric intervention and that dural puncture epidural analgesia would show superior analgesic performance relative to standard epidural analgesia without compromising obstetric or neonatal outcomes.

Dural puncture epidural analgesia has emerged as a modification of the standard epidural technique. In this approach, the epidural space is identified in the usual manner, and the dura is intentionally punctured with a fine spinal needle to confirm midline placement and create a small dural conduit, but no intrathecal medication is administered. This technique is intended to improve transmeningeal spread of epidurally administered local anesthetic and opioid, thereby accelerating analgesia onset, enhancing sacral blockade, reducing asymmetric block, and decreasing the need for rescue boluses [3,4,5,6]. A systematic review and meta-analysis of randomized controlled trials reported that dural puncture epidural analgesia increased the proportion of women achieving satisfactory pain relief within the first 10–20 min compared with conventional epidural analgesia, without evidence of increased maternal or fetal adverse events [3].

Subsequent randomized studies and meta-analytic evidence have refined this interpretation by examining dural puncture epidural analgesia in combination with modern maintenance strategies. Trials using programmed intermittent epidural bolus and low-concentration local anesthetic regimens have generally shown faster onset of analgesia, improved sacral coverage, lower top-up requirements, and fewer asymmetric blocks with dural puncture epidural analgesia compared with conventional epidural analgesia [4,5,6]. However, the magnitude and consistency of benefit appear to depend on several technical and clinical factors, including spinal needle gauge, catheter function, local anesthetic concentration, maintenance regimen, parity, and patient population. Recent data in obese parturients and nulliparous laboring women suggest that the superiority of dural puncture epidural analgesia may not be universal and may be more evident for block quality and sacral spread than for global analgesic success or clinical obstetric outcomes [7,8,9].

Whether the analgesic advantages of dural puncture epidural analgesia translate into meaningful differences in labor progression, fetal head position, obstetric intervention rates, or neonatal outcomes remains uncertain. Most available studies have focused primarily on analgesia quality, onset time, or block characteristics, whereas comparative data on labor dynamics and neonatal outcomes are more limited. Moreover, studies comparing dural puncture epidural analgesia with standard epidural analgesia often do not include a no-epidural control group, making it difficult to separate technique-specific effects from the broader influence of neuraxial analgesia and from obstetric confounding. Contemporary population-level data suggest that labor epidural analgesia is not independently associated with clinically important adverse neonatal outcomes after robust adjustment for confounding, but residual uncertainty persists for specific biochemical outcomes such as umbilical cord pH and for selected obstetric endpoints [10].

Therefore, the present study aimed to compare dural puncture epidural analgesia, standard epidural analgesia, and no-epidural labor in terms of labor progression, fetal head position, obstetric intervention rates, analgesia quality, and neonatal outcomes. Because observational comparisons in labor analgesia are particularly vulnerable to baseline imbalance and confounding, we applied complementary statistical approaches, including multivariable adjustment, quantile regression, penalized logistic regression for rare outcomes, and propensity-score-based weighting. We hypothesized that, when contemporary low-dose neuraxial regimens are used and major confounders are accounted for, neither standard epidural nor dural puncture epidural analgesia would be associated with prolongation of labor or increased obstetric intervention rates, while dural puncture epidural analgesia might demonstrate improved analgesia quality compared with standard epidural analgesia.

### Objectives

The primary objective of this study was to compare dural puncture epidural analgesia, standard epidural analgesia, and labor without epidural analgesia with respect to labor progression, with particular emphasis on the duration of the second stage of labor.

Secondary objectives were to compare the three groups in terms of first-stage duration, fetal head position, cesarean delivery, instrumental vaginal delivery, blood loss, transfusion requirement, and neonatal outcomes, including 1 min and 5 min Apgar scores, umbilical venous pH, biochemical acidosis, recorded fetal acidosis, birth weight, neonatal intensive care unit admission, neonatal resuscitation, and phototherapy. Among women who received neuraxial labor analgesia, additional secondary objectives were to compare standard epidural and dural puncture epidural analgesia with respect to analgesia quality and maternal side effects, including pain scores, rescue bolus requirement, hypotension, motor block, total local anesthetic dose, and adverse effects.

## 2. Materials and Methods

### 2.1. Study Design and Setting

This was a single-center, retrospective, observational cohort study conducted at the Department of Obstetrics and Gynecology, SBU Gazi Yasargil Training and Research Hospital. Medical records of women who delivered between 1 January 2024 and 1 February 2026 were reviewed.

The study protocol was approved by the Clinical Research Ethics Committee of Gazi Yaşargil Training and Research Hospital, Diyarbakır, Türkiye (Protocol No. KAD-FR-01; approval date: 12 February 2026). Because of the retrospective design, anonymized data collection, and absence of direct patient contact, the requirement for written informed consent was waived by the ethics committee.

Data were obtained from the hospital information management system, anesthesia procedure records, obstetric follow-up forms, and neonatal medical files. All data were anonymized before being transferred to the study database. No direct patient contact was made, and no additional intervention was performed for the purpose of the study.

The study was designed to compare labor progression, obstetric intervention rates, analgesia-related outcomes, and neonatal outcomes among women who delivered without epidural analgesia, women who received standard epidural analgesia, and women who received dural puncture epidural analgesia.

### 2.2. Inclusion and Exclusion Criteria

Women aged 18–45 years with term, singleton, vertex-presenting pregnancies and an American Society of Anesthesiologists physical status of I–III were considered eligible. Women with available obstetric, anesthetic, and neonatal data sufficient for assessment of the study outcomes were included in the final analysis.

Patients were excluded if they had multiple gestations, previous cesarean delivery, breech or transverse fetal lie, placenta previa, placental abruption, major obstetric complications, or records that did not allow assessment of the main study outcomes. Accidental dural puncture during standard epidural catheter placement and unsuccessful neuraxial block were also considered exclusion criteria for the epidural groups.

### 2.3. Study Population and Grouping

During the study period, 168 eligible women who met the predefined inclusion criteria constituted the final analytical cohort. Patients were grouped according to the labor analgesia technique documented in clinical records.

Women who delivered without neuraxial labor analgesia were included in the no-epidural control group (*n* = 58). Women who received conventional epidural analgesia without intentional dural puncture were included in the standard epidural analgesia group (*n* = 56). Women who received dural puncture epidural analgesia without intrathecal drug administration were included in the dural puncture epidural group (*n* = 54).

Because this was a retrospective cohort study, group allocation was not randomized. Instead, patients were assigned to study groups according to the analgesia technique actually received during labor. Women who did not receive neuraxial analgesia were retained as a pragmatic no-epidural control group, rather than being matched one-to-one to the epidural groups, because the study aimed to capture real-world practice across the full spectrum of labor analgesia decisions at our institution and because a matched design would have discarded a substantial proportion of the eligible cohort, further reducing power for the primary outcome. The choice of analgesia technique, including the decision to forgo neuraxial analgesia, was made by the patient and the attending obstetric team on clinical grounds rather than by protocol, so women who declined or did not request epidural analgesia may systematically differ from those who did (for example, in pain tolerance, labor progression at presentation, or parity). This introduces a clear potential for selection bias and confounding by indication that cannot be fully removed by statistical adjustment; we address this explicitly by combining multivariable adjustment, quantile regression, propensity-score weighting, and a nulliparous sensitivity analysis and by discussing residual confounding as a limitation. Given the potential for confounding by indication in epidural labor analgesia research, adjusted and propensity-based approaches were considered essential for interpretation [11].

### 2.4. Neuraxial Analgesia and Follow-Up Protocols

All neuraxial procedures were performed by anesthesiologists experienced in obstetric anesthesia, according to the institutional labor analgesia protocol. Before the procedure, intravenous access was established in all patients receiving neuraxial analgesia. Non-invasive blood pressure, heart rate, and peripheral oxygen saturation were monitored as part of routine clinical care. Fetal heart rate monitoring was continued by cardiotocography throughout labor according to routine obstetric practice.

All neuraxial procedures were performed under aseptic conditions. Skin antisepsis was achieved using 2% chlorhexidine alcohol. Patients were positioned in either the sitting or lateral decubitus position according to clinical circumstances and operator preference. Apart from the neuraxial technique itself, obstetric monitoring, analgesia follow-up, maternal surveillance, and fetal monitoring were performed according to the same institutional protocol in both epidural groups.

Analgesia was initiated and maintained using the same institutional low-dose bupivacaine–fentanyl regimen in both epidural groups; the two epidural groups differed only by the intentional dural puncture step in the dural puncture epidural group.

#### 2.4.1. Standard Epidural Analgesia Protocol

Standard epidural analgesia was performed according to the institutional labor analgesia protocol. The aim of neuraxial labor analgesia was to provide sensory blockade from T10 to S5 with minimal motor block. Cardiotocographic fetal monitoring was continued during neuraxial catheter placement, and all procedures were performed under strict aseptic conditions.

The epidural space was identified at the L3–L4 or L4–L5 interspace using an 18-gauge Tuohy needle and the loss-of-resistance-to-saline technique. If the L2–L3 interspace was used because of clinical or technical considerations, only a standard epidural technique was performed. After identification of the epidural space, a 19-gauge epidural catheter was advanced 3–5 cm into the epidural space. In obese patients, 5–6 cm of catheter was left in the epidural space when required. The catheter was fixed with a transparent sterile dressing after the patient resumed a normal sitting position, and the insertion site was kept visible.

Before initiation of analgesia, catheter position was assessed using standard institutional checks, including inspection for free flow of cerebrospinal fluid or blood, gentle aspiration with a 3 mL syringe, and administration of a test dose. A conventional test dose was not mandatory. Instead, 5 mL of the epidural solution containing 0.1% bupivacaine with fentanyl 2 micrograms/mL was used. Profound motor block was considered suggestive of intrathecal catheter placement. When intravascular placement was suspected, lidocaine 1 mg/kg, up to a maximum dose of 80 mg, was used as a test dose while monitoring for symptoms such as circumoral tingling or tinnitus.

Analgesia was initiated with an epidural top-up dose of 15–20 mL of 0.1% bupivacaine with fentanyl 2 micrograms/mL. Maintenance analgesia was provided according to the institutional protocol using either continuous epidural infusion with patient-controlled epidural analgesia or programmed intermittent epidural bolus and patient-controlled epidural analgesia. For continuous infusion with patient-controlled epidural analgesia, the basal infusion rate was 0–10 mL/h, with a patient-controlled bolus of 10 mL and a 20 min lockout interval. For programmed intermittent epidural bolus, a programmed bolus of 10 mL was administered every 45 min, with a patient-controlled bolus of 5 mL and a 10 min lockout interval.

Additional rescue or top-up boluses were administered when clinically required, according to maternal pain scores and adequacy of sensory blockade. Maternal blood pressure was monitored every 5 min for the first 20 min after initiation of the block and every 30 min thereafter. Sensory block level was assessed 20 min after initiation and every 2 h thereafter. Motor block was assessed 20 min after initiation and every 4 h thereafter. Fetal heart rate monitoring was continued throughout labor according to routine obstetric practice.

#### 2.4.2. Dural Puncture Epidural Analgesia Protocol

Dural puncture epidural analgesia was performed using the same institutional epidural analgesia protocol, with the addition of intentional dural puncture before epidural catheter placement. The epidural space was identified at the L3–L4 or L4–L5 interspace using an 18-gauge Tuohy needle and the loss-of-resistance-to-saline technique under strict aseptic conditions. Cardiotocographic fetal monitoring was continued during neuraxial catheter placement.

After localization of the epidural space, a 27-gauge spinal needle was advanced through the Tuohy needle until free cerebrospinal fluid flow was observed. The 27-gauge needle was selected, in line with institutional practice and prior randomized evidence, to minimize the risk of post-dural puncture headache while still permitting reliable confirmation of dural puncture and transmeningeal drug spread; because needle gauge is a recognized technical determinant of the analgesic effect of dural puncture epidural analgesia, this choice should be considered when comparing our findings with studies using finer or coarser spinal needles. No intrathecal local anesthetic or opioid was administered. After confirmation of cerebrospinal fluid return, the spinal needle was withdrawn, and a 19-gauge epidural catheter was advanced 3–5 cm into the epidural space. In obese patients, 5–6 cm of catheter was left in the epidural space when required. The catheter was fixed with a transparent sterile dressing after the patient resumed a normal sitting position, and the insertion site was kept visible.

Catheter position was assessed according to the same institutional checks used for standard epidural analgesia, including inspection for free flow of cerebrospinal fluid or blood, gentle aspiration with a 3 mL syringe, and administration of a test dose. A 5 mL epidural test dose of 0.1% bupivacaine with fentanyl 2 micrograms/mL was used. Profound motor block was considered suggestive of intrathecal catheter placement. When intravascular placement was suspected, lidocaine 1 mg/kg, up to a maximum of 80 mg, was used while monitoring symptoms of intravascular local anesthetic administration.

Analgesia was initiated with an epidural top-up dose of 15–20 mL of 0.1% bupivacaine with fentanyl 2 micrograms/mL. Maintenance analgesia was provided using the same regimen as in the standard epidural group. According to the institutional protocol, this included either continuous epidural infusion with patient-controlled epidural analgesia, using a basal infusion rate of 0–10 mL/h and a 10 mL patient-controlled bolus with a 20 min lockout interval, or programmed intermittent epidural bolus with patient-controlled epidural analgesia, using a programmed bolus of 10 mL every 45 min and a 5 mL patient-controlled bolus with a 10 min lockout interval.

Rescue or top-up bolus administration, maternal hemodynamic monitoring, and sensory and motor block assessment followed the same schedule described for the standard epidural group (Section 2.4.1), and fetal heart rate monitoring was continued throughout labor according to routine obstetric practice.

The dural puncture epidural technique differed from combined spinal–epidural analgesia in that no intrathecal medication was administered. Therefore, all analgesic dosing was delivered through the epidural catheter, using the same low-dose bupivacaine–fentanyl regimen as in the standard epidural group.

### 2.5. Study Variables and Outcome Measures

The primary outcome was the duration of the second stage of labor. Secondary outcomes included the duration of the first stage of labor, active pushing time, fetal head position, mode of delivery, obstetric intervention rates, analgesia-related outcomes, maternal physiological events, and neonatal outcomes. Second-stage duration was defined as the interval from complete cervical dilation to delivery, and active pushing time was defined as the interval from the onset of active maternal pushing to delivery; these were recorded as distinct variables.

Maternal demographic and baseline variables included age, body mass index, parity, gestational age at delivery, initial cervical dilation, estimated fetal weight, and use of labor induction or augmentation. Gestational age was converted into decimal weeks when recorded in weeks–plus–days format; because it was documented in only a subset of records, it was not used in the adjusted models. These variables were selected because of their potential influence on labor progression, mode of delivery, and neonatal outcomes.

Labor and obstetric outcomes included first-stage duration, second-stage duration, active pushing time, fetal head position, mode of delivery, blood loss, and transfusion requirement. Fetal head position was categorized as occiput anterior, occiput posterior, or asynclitic when documented in the obstetric records. Mode of delivery was categorized as spontaneous vaginal delivery, instrumental vaginal delivery, or cesarean delivery.

Analgesia-related variables were evaluated only in women who received neuraxial labor analgesia. These included the type of epidural technique, local anesthetic regimen, total local anesthetic dose, rescue or top-up bolus requirement, pain score, motor block, hypotension, and maternal side effects. Pain intensity was assessed using the visual analog scale when documented. Motor block was assessed using the Bromage scale. Hypotension was defined as a decrease of at least 20% in mean arterial pressure from baseline after neuraxial analgesia initiation.

Neonatal and laboratory outcomes included 1 min and 5 min Apgar scores, umbilical cord blood venous pH, biochemical acidosis, recorded fetal acidosis, birth weight, neonatal intensive care unit admission, need for neonatal resuscitation, and phototherapy. Biochemical acidosis was defined as an umbilical cord venous pH value < 7.20. Umbilical venous, rather than arterial, cord blood gas sampling was used because venous sampling was the consistent institutional practice throughout the study period and paired arterial sampling was not routinely available in the retrospective records; venous pH is less sensitive than arterial pH to acute intrapartum hypoxic events, and this should be considered a limitation when comparing our biochemical acidosis rates with studies using arterial cord gases. Recorded fetal acidosis was defined as fetal acidosis documented as such in the obstetric or neonatal medical record by the attending clinician; unlike biochemical acidosis, this is a documentation-based clinical judgment rather than a value derived from a fixed laboratory threshold, and no single standardized diagnostic criterion underlies it across records. Neonatal morbidity in this study was operationalized through its component outcomes (Apgar scores, neonatal intensive care unit admission, neonatal resuscitation, and phototherapy) rather than as a single composite variable. Implausible cord venous pH values attributable to data-entry errors were checked against the original records and corrected when appropriate before analysis.

### 2.6. Sample Size Calculation

The sample size was determined based on the primary outcome, which was the duration of the second stage of labor. For a three-group comparison, assuming a moderate effect size (Cohen’s f = 0.25), an alpha level of 0.05, and 80% statistical power, the minimum required sample size was calculated as 159 women in total, corresponding to approximately 53 women per group. Because second-stage duration was non-normally distributed, the corresponding between-group comparison was performed with the Kruskal–Wallis test; the parametric effect-size assumption was used only for sample size estimation.

During the study period from 1 January 2024 to 1 February 2026, a total of 168 eligible women who met the predefined inclusion criteria were identified and included in the final analysis. Therefore, the final analytical cohort exceeded the minimum sample size required by the power analysis and was considered adequate for the primary endpoint. The final study groups consisted of 58 women in the no-epidural control group, 56 women in the standard epidural group, and 54 women in the dural puncture epidural group.

### 2.7. Statistical Analysis

Statistical analyses were performed using Python version 3.14 with the pandas version 3.0.3, NumPy version 2.5.2, SciPy version 1.180, statsmodels version 0.14.6, and scikit-posthocs version 0.14.0 libraries. The distribution of continuous variables was assessed using the Shapiro–Wilk test and visual inspection of histograms and quantile-quantile plots. Normally distributed continuous variables are presented as mean ± standard deviation, whereas non-normally distributed variables are presented as median and interquartile range. Categorical variables are presented as numbers and percentages. Missing data were not imputed; variables with substantial missingness, most notably gestational age, were reported descriptively and excluded from adjusted models, while all other variables used in group comparisons and adjusted analyses were available for the full analytic cohort. Cases with missing values for a given outcome were excluded from that specific comparison on a variable-by-variable (complete-case) basis.

For comparisons among the three groups, one-way analysis of variance was used for normally distributed continuous variables, and the Kruskal–Wallis test was used for non-normally distributed continuous variables. Categorical variables were compared using the chi-square test or Fisher’s exact test, as appropriate. When statistically significant differences were identified, post hoc comparisons were performed using Dunn–Bonferroni testing for continuous variables and pairwise Fisher exact testing for categorical variables.

Because of the observational design and baseline imbalance among groups, additional adjusted analyses were performed, consistent with contemporary propensity-score approaches used to reduce confounding in epidural labor analgesia studies [11]. Log-transformed labor-stage durations were analyzed using analysis of covariance with adjustment for maternal age, parity, and initial cervical dilation. Gestational age was not included in the adjusted models because it was documented in only a subset of records. Quantile regression was used to evaluate whether the epidural technique was associated with prolongation of the upper percentiles of second-stage duration. This approach allowed assessment of potential effects not only on central tendency but also on the upper tail of the distribution.

For rare binary outcomes and outcomes with zero events in one group, Firth penalized logistic regression was used to reduce small-sample bias and address separation. Propensity-score-based inverse probability of treatment weighting was specifically applied to the comparison between the standard epidural and dural puncture epidural analgesia groups. Covariate balance after weighting was evaluated using standardized mean differences. A sensitivity analysis restricted to nulliparous women was also performed to explore the influence of parity on labor-stage duration.

A two-sided *p*-value < 0.05 was considered statistically significant.

This manuscript was prepared in accordance with the Strengthening the Reporting of Observational Studies in Epidemiology (STROBE) guidelines, and the completed STROBE checklist is provided as Supplementary Material.

## 3. Results

### 3.1. Study Population and Baseline Characteristics

**Figure 1:**
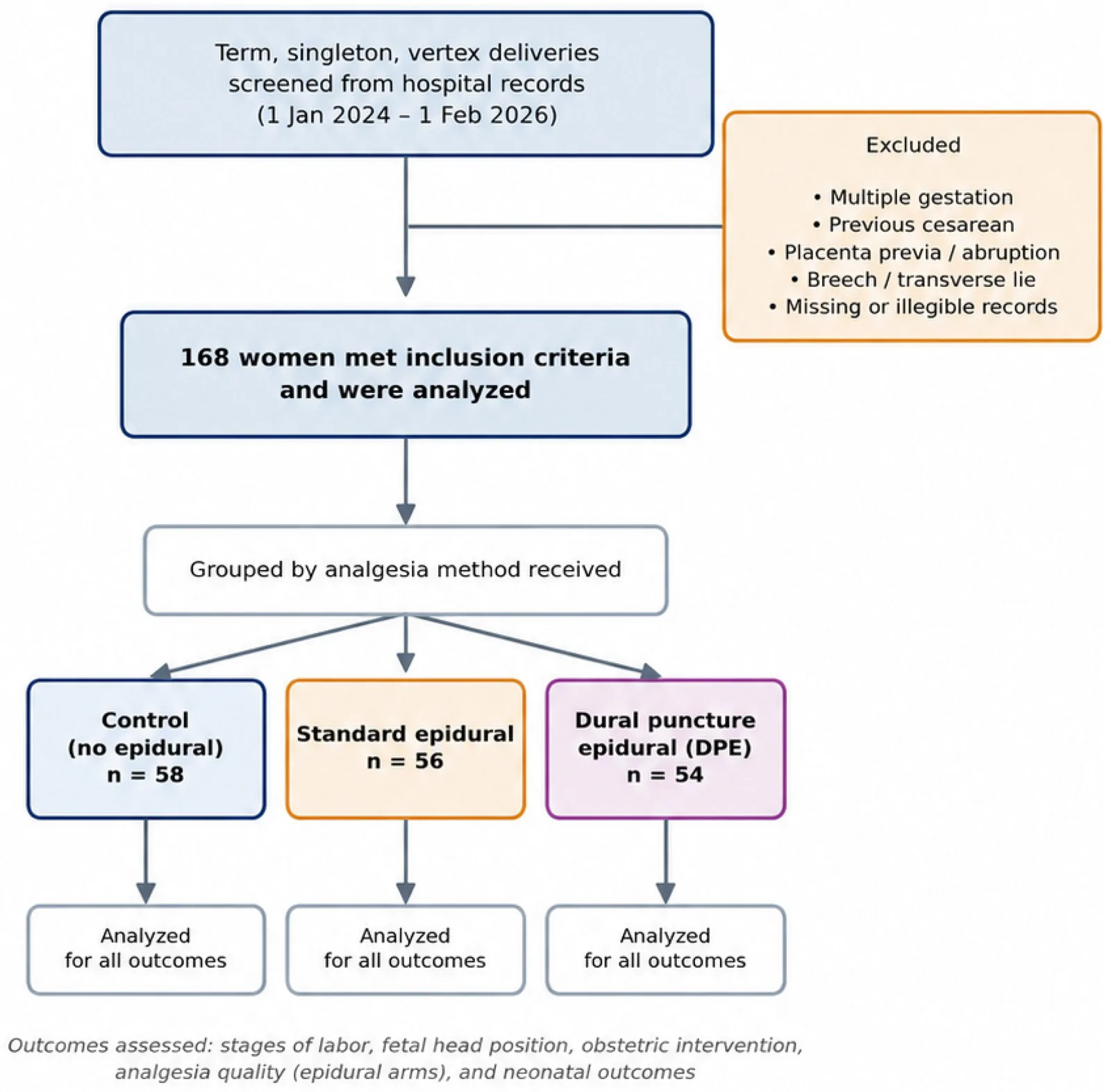
Study flow diagram. The screening, inclusion, and exclusion of cases, their allocation to groups and the number of cases analyzed as shown.

A total of 168 women were included in the final analysis. The no-epidural control group included 58 women, the standard epidural analgesia group included 56 women, and the dural puncture epidural analgesia group included 54 women (Figure 1).

Baseline demographic and obstetric characteristics differed among the groups (Table 1). Women in the control group were older than those in the epidural groups, with a median age of 27.0 years compared with 23.0 years in the standard epidural group and 24.0 years in the dural puncture epidural group (*p* = 0.003). Parity also differed significantly across groups; the control group had higher parity than both epidural groups, whereas the standard epidural and dural puncture epidural analgesia groups were predominantly nulliparous (*p* < 0.001). Initial cervical dilation was greater in the control group than in the epidural groups, with median values of 3.0 cm, 2.0 cm, and 2.0 cm, respectively (*p* < 0.001). Induction or augmentation of labor was more frequent in the epidural groups than in the control group (79.3%, 98.2%, and 94.4%, respectively; *p* = 0.001). Body mass index and estimated fetal weight were similar among groups. Gestational age, which was documented in only a subset of records, did not differ significantly among groups (*p* = 0.057). These baseline differences indicated substantial imbalance among groups, particularly in parity, initial cervical dilation, maternal age, and induction or augmentation status. Because the epidural groups were predominantly nulliparous and had higher induction or augmentation rates, adjusted analyses were essential to reduce confounding by indication.

### 3.2. Labor Progression and Obstetric Outcomes

**Figure 2:**
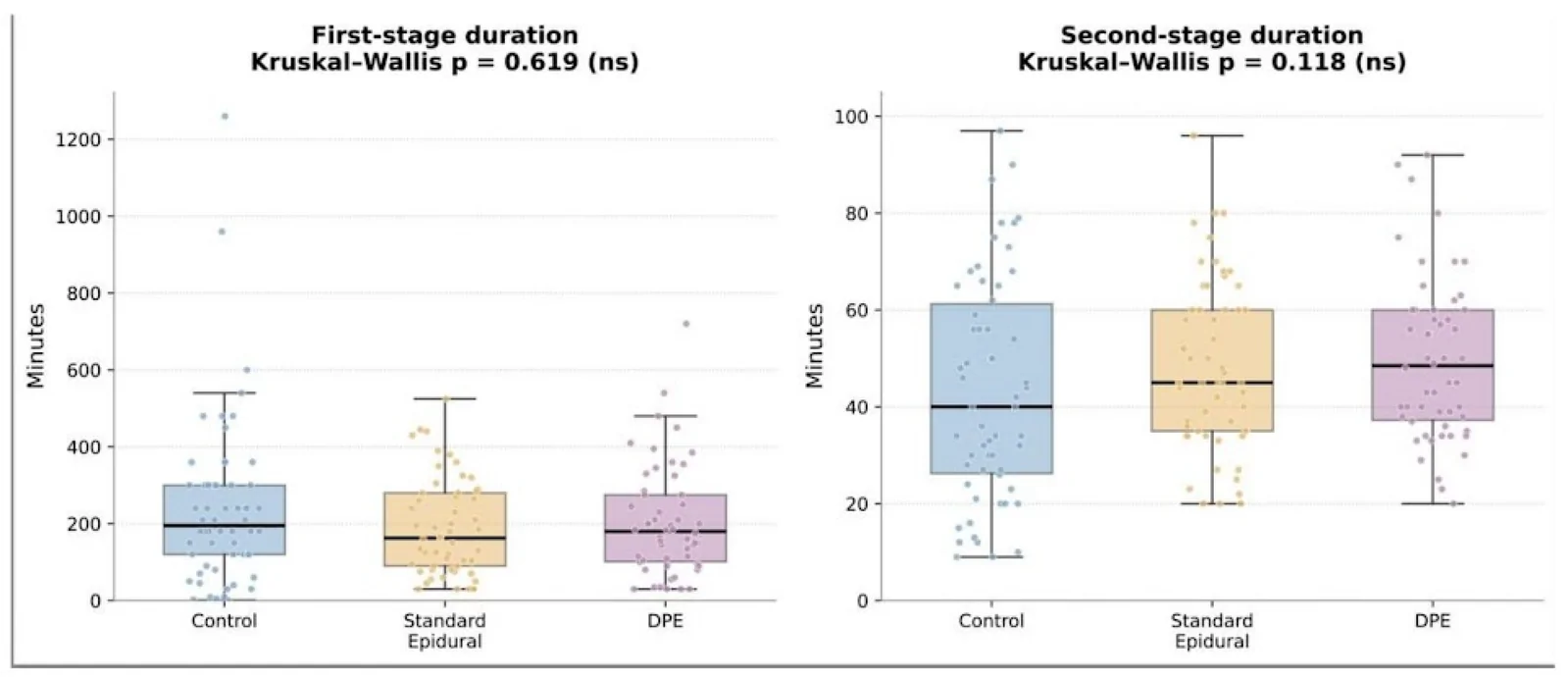
Distribution of first- and second-stage labor durations by group (box plots with individual observations). The between-group differences are not statistically significant; overall Krushkal-Wallis *p*-values are annotated on each panel.

The primary outcome, duration of the second stage of labor, was comparable across the three groups. Median second-stage duration was 40.0 min in the control group, 45.0 min in the standard epidural group, and 48.5 min in the dural puncture epidural group (*p* = 0.118). Similarly, first-stage duration was comparable among groups, with median values of 195 min, 163 min, and 180 min, respectively (*p* = 0.619). Active pushing time, defined as the interval from the onset of active maternal pushing (not from full cervical dilation) to delivery, also remained comparable, with median values of 8.0 min in the control group, 8.5 min in the standard epidural group, and 12.0 min in the dural puncture epidural group (*p* = 0.292, Figure 2).

Blood loss tended to be higher in the epidural groups than in the control group, but the difference did not reach statistical significance. Median blood loss was 450 mL in the control group and 540 mL in both epidural groups (*p* = 0.091). Transfusion requirements were low and similar among the three groups (3.4%, 3.6%, and 1.9%, respectively; *p* = 0.840).

Cesarean delivery rates were low and did not differ significantly among groups (Table 2). Cesarean delivery occurred in one woman in the control group (1.7%), two women in the standard epidural group (3.6%), and one woman in the dural puncture epidural group (1.9%) (*p* = 0.773). Instrumental vaginal delivery was rare, with only one case observed in the control group and none in either epidural group (*p* = 0.385). No occiput posterior fetal head position was documented in the available records; therefore, this outcome was addressed descriptively and could not be statistically compared.

**Figure 3:**
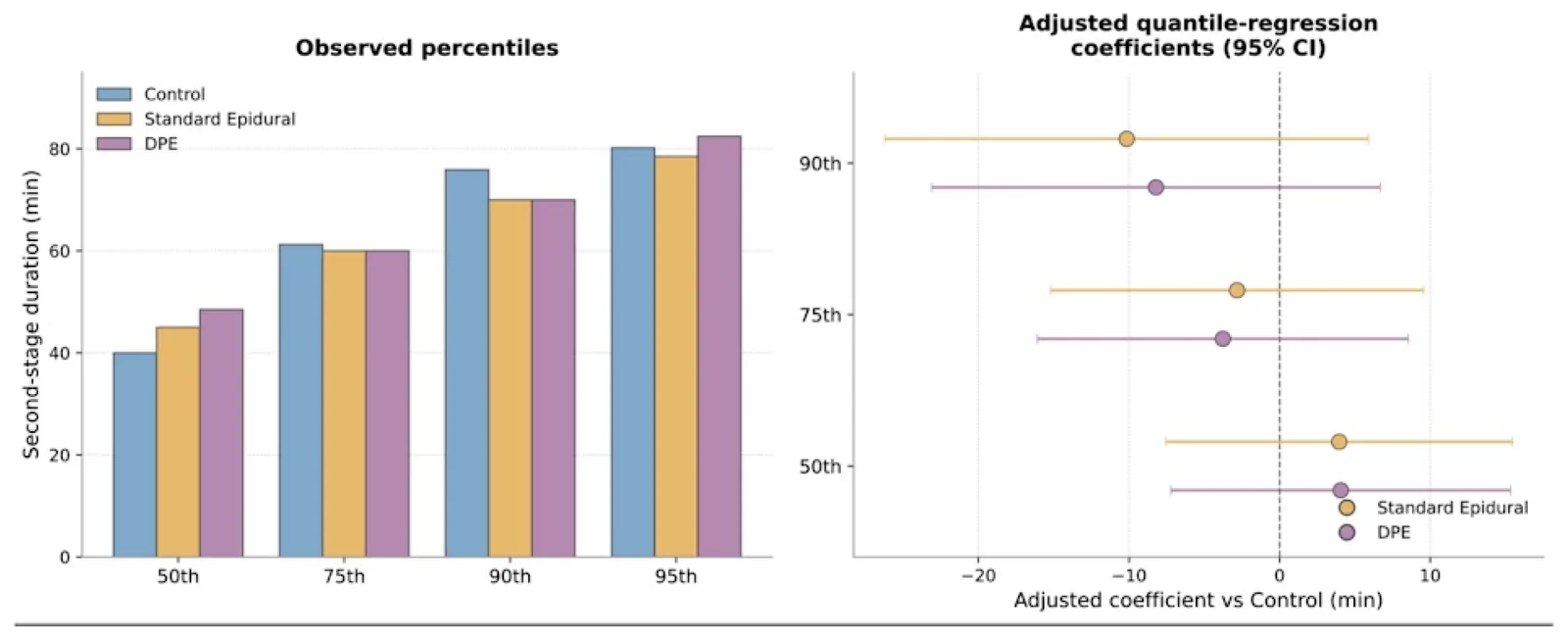
Upper-percentile and adjusted quantile regression analysis of second-stage duration. (**Left**): observed percentiles (50/75/90/95th) of second-stage duration by group. (**Right**): adjusted quantile-regression coefficients relative to control with 95% confidence intervals. No epidural-related prolongation is seen at the upper percentiles (all Cis include 0).

To evaluate whether epidural analgesia was associated with prolongation of the upper part of the second-stage duration distribution, percentile analysis and adjusted quantile regression were performed (Figure 3). No epidural-related prolongation was observed at the 50th, 75th, or 90th percentiles. At the upper percentiles, the adjusted coefficients for both epidural groups were in the direction of shorter rather than longer duration, and all confidence intervals included zero. These findings did not support an epidural-related upper-percentile prolongation effect in this cohort.

### 3.3. Neonatal Outcomes

One-minute and five-minute Apgar scores were similar across the three groups. The median 1 min Apgar score was 8 in all groups (*p* = 0.416), and the median 5 min Apgar score was 9 in all groups (*p* = 0.756). No neonate required resuscitation.

Umbilical cord venous pH did not differ significantly among groups. Median cord pH was 7.380 in the control group, 7.367 in the standard epidural group, and 7.370 in the dural puncture epidural group (*p* = 0.266). Biochemical acidosis, defined as cord venous pH < 7.20, occurred in no neonates in the control group, one neonate in the standard epidural group (1.8%), and two neonates in the dural puncture epidural group (3.7%) (*p* = 0.335). In contrast, recorded fetal acidosis was the only neonatal endpoint that differed significantly between groups, with rates of 0%, 7.1% (4/56), and 16.7% (9/54), respectively (*p* = 0.004).

Neonatal intensive care unit admission was numerically more frequent in the epidural groups, occurring in 5.2% of neonates in the control group, 10.7% in the standard epidural group, and 16.7% in the dural puncture epidural group; however, this difference was not statistically significant (*p* = 0.145). Birth weight did not differ among the groups (*p* = 0.389). Phototherapy was administered only in the control group, and the between-group difference approached but did not reach statistical significance (*p* = 0.055, Table 3).

**Figure 4:**
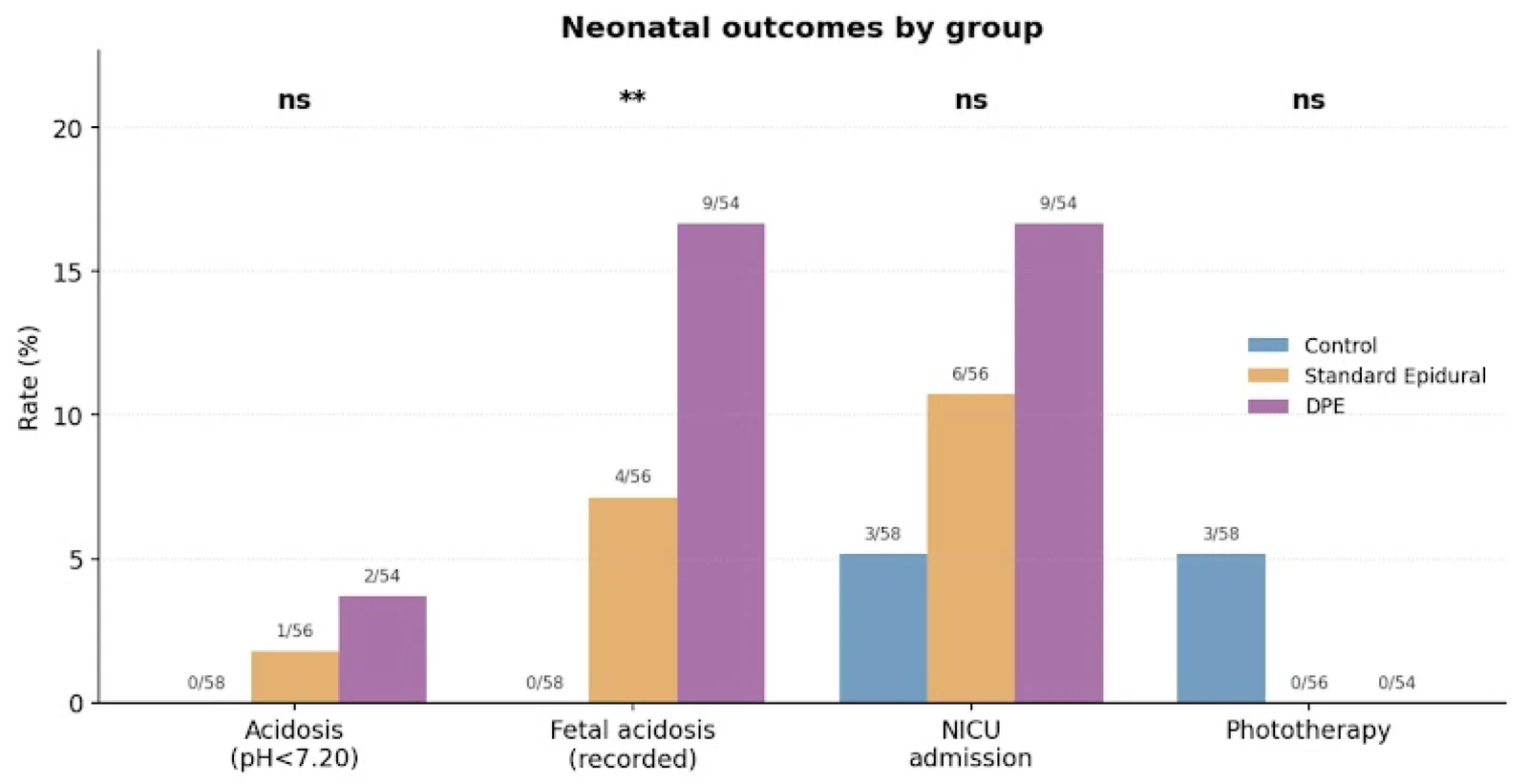
Rates of neonatal outcomes by group. Rates of neonatal outcomes by group (acidosis [cord pH < 7.20], recorded fetal acidosis, NICU admission, phototherapy). Event/total counts are shown above the bars; significance markers above each outcome display the three-group result (** *p* < 0.01, ns = not significant).

Objective neonatal measures were reassuring; umbilical cord pH and biochemical acidosis did not differ significantly among groups, Apgar scores were normal in all groups, and no neonate required resuscitation (Figure 4). The only significant between-group difference was in the recorded fetal acidosis flag, which was not corroborated by the objective cord-pH values and was not accompanied by lower Apgar scores, neonatal resuscitation, or a statistically significant increase in neonatal intensive care unit admission.

### 3.4. Analgesia-Related Outcomes in the Epidural Groups

Analgesia-related outcomes were assessed only in women who received neuraxial labor analgesia. Pain scores were numerically lower in the dural puncture epidural group than in the standard epidural group, but the difference was not statistically significant. The median visual analog scale score was 1 in the standard epidural group and 0 in the dural puncture epidural group (*p* = 0.210).

**Figure 5:**
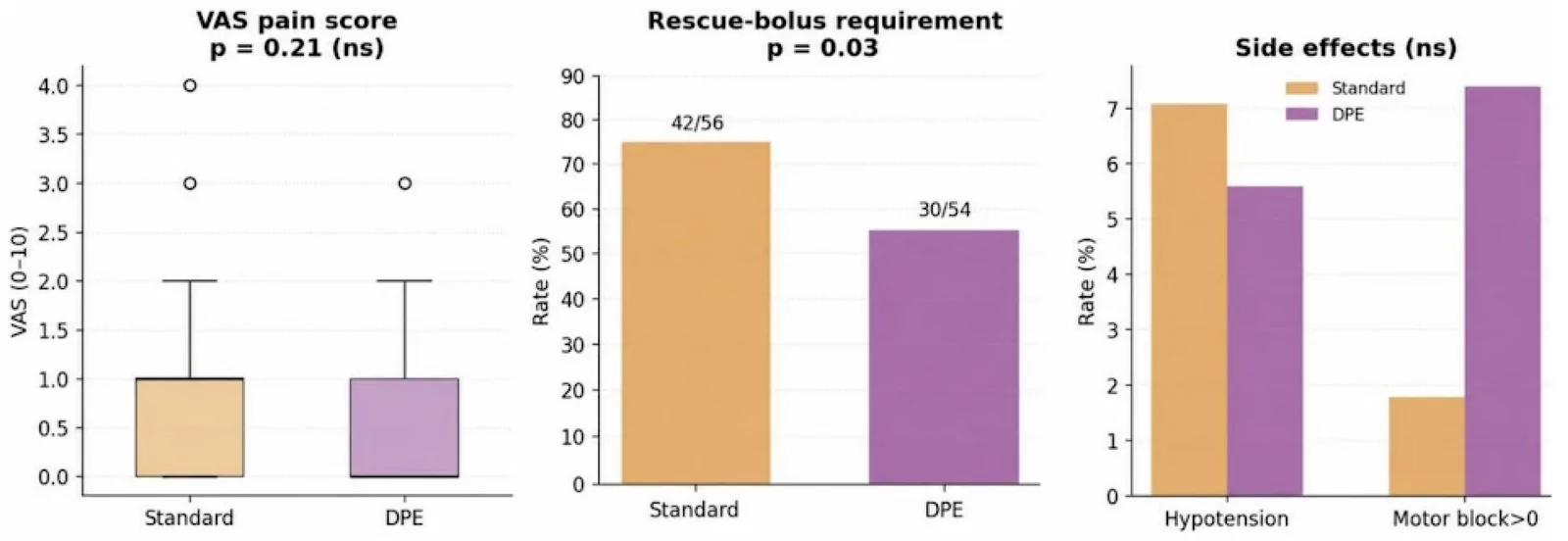
Indicators of analgesia quality in the standard epidural and dural puncture epidural analgesia groups. The VAS pain score is shown on the left, the rescue bolus requirement in the middle, and hypotension and motor-block rates on the right. The rescue bolus requirement was lower with dural puncture epidural analgesia, but the difference was not statistically significant (*p* = 0.083), whereas the VAS pain score and side-effect rates were also not significantly different.

The rescue or top-up bolus requirement was lower in the dural puncture epidural group, but the difference did not reach statistical significance. Top-up boluses were required in 39 of 56 women in the standard epidural group (69.6%) and in 30 of 54 women in the dural puncture epidural group (55.6%) (*p* = 0.183). Hypotension occurred in 7.1% of women in the standard epidural group and 5.6% of women in the dural puncture epidural group. Motor block was uncommon in both groups, with only isolated cases recorded, including a single high Bromage value in the dural puncture epidural group. Overall, these findings suggest a trend toward improved analgesic performance with dural puncture epidural analgesia, mainly reflected by a lower rescue bolus requirement, although the difference was not statistically significant in this cohort (Figure 5).

### 3.5. Adjusted Analyses for Labor Duration and Biochemical Acidosis

Because of baseline imbalance among groups, adjusted analyses were performed to ensure that observed associations were not driven primarily by baseline differences between groups. In the analysis of covariance model adjusted for maternal age, parity, and initial cervical dilation, neither epidural technique was associated with a shorter second stage: the standard epidural group did not differ significantly from the control group (beta = +0.19; *p* = 0.066), whereas the dural puncture epidural group showed a small adjusted increase in log-transformed second-stage duration (beta = +0.23; *p* = 0.022). Gestational age was not included in the adjusted models because it was documented in only a subset of records. Parity was the dominant determinant of second-stage duration in the model.

For the first-stage duration, adjusted estimates were strongly influenced by initial cervical dilation. Adjusted quantile regression showed no epidural-related prolongation at the median, 75th percentile, or 90th percentile, with all confidence intervals including zero; at the upper percentiles, the coefficients for both epidural groups were in the direction of shorter duration. Taken together with the comparable crude durations and the nulliparous sensitivity analysis, these findings do not provide robust evidence of epidural-related second-stage prolongation, although the adjusted mean model showed a small dural-puncture-associated increase that was not consistent across analytic methods.

Because only three biochemical acidosis events occurred in the entire cohort, and none in the control group, Firth penalized logistic regression was used to reduce small-sample bias and address separation. In the adjusted model for cord venous pH < 7.20, the odds ratio relative to the control group was approximately 3.16 for standard epidural analgesia and 5.57 for dural puncture epidural analgesia; both estimates had very wide confidence intervals that crossed the null value and were not statistically significant. The small number of events and the zero-event control group precluded a stable estimate of risk (Table 4).

**Figure 6:**
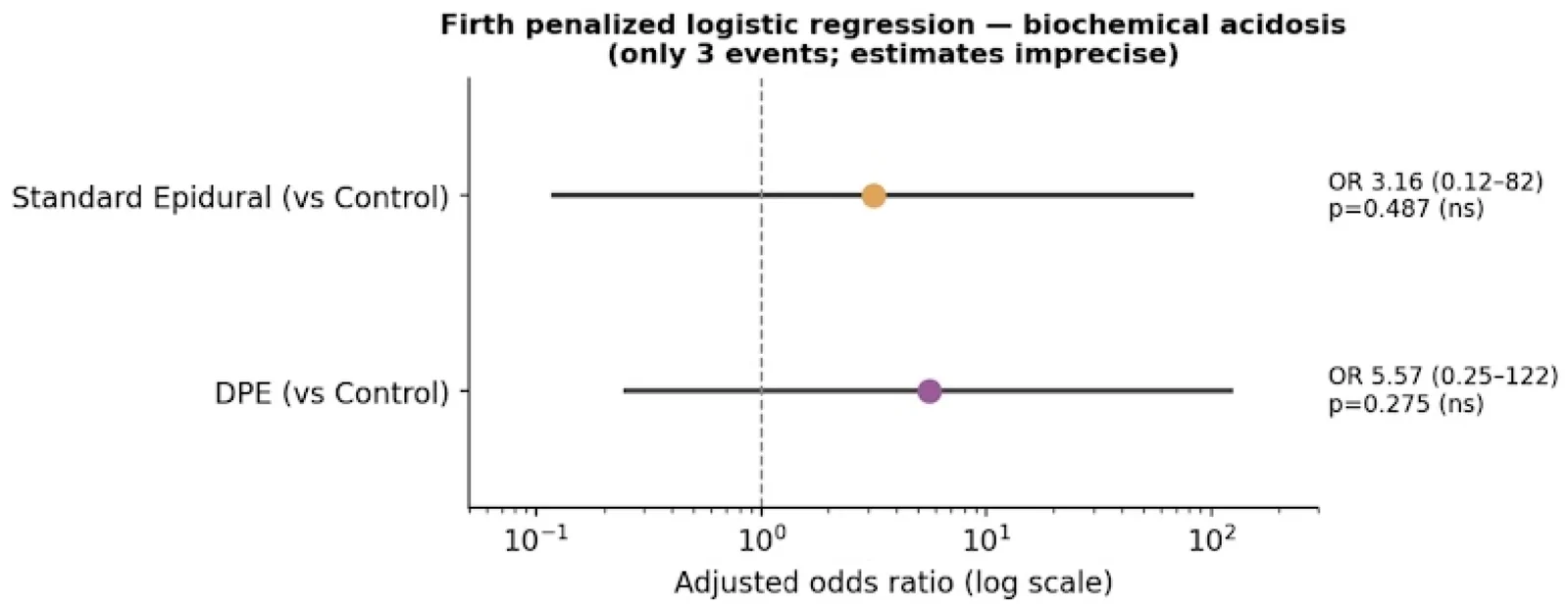
Forest plot of adjusted odds ratios from the Firth penalized logistic regression model for biochemical acidosis.Ors (95% CI) and significance status (ns = not significant) are annotated on the right. The very wide intervals reflect the impression created by the small number of events and the zero-event control arm.

The forest plot of the Firth penalized logistic regression model visually confirmed the imprecision of the estimated associations (Figure 6). The point estimates for both epidural groups were elevated, but the confidence intervals were wide and crossed the null value, particularly because of the zero-event control group.

### 3.6. Propensity-Score Weighting and Nulliparous Sensitivity Analysis

**Figure 7:**
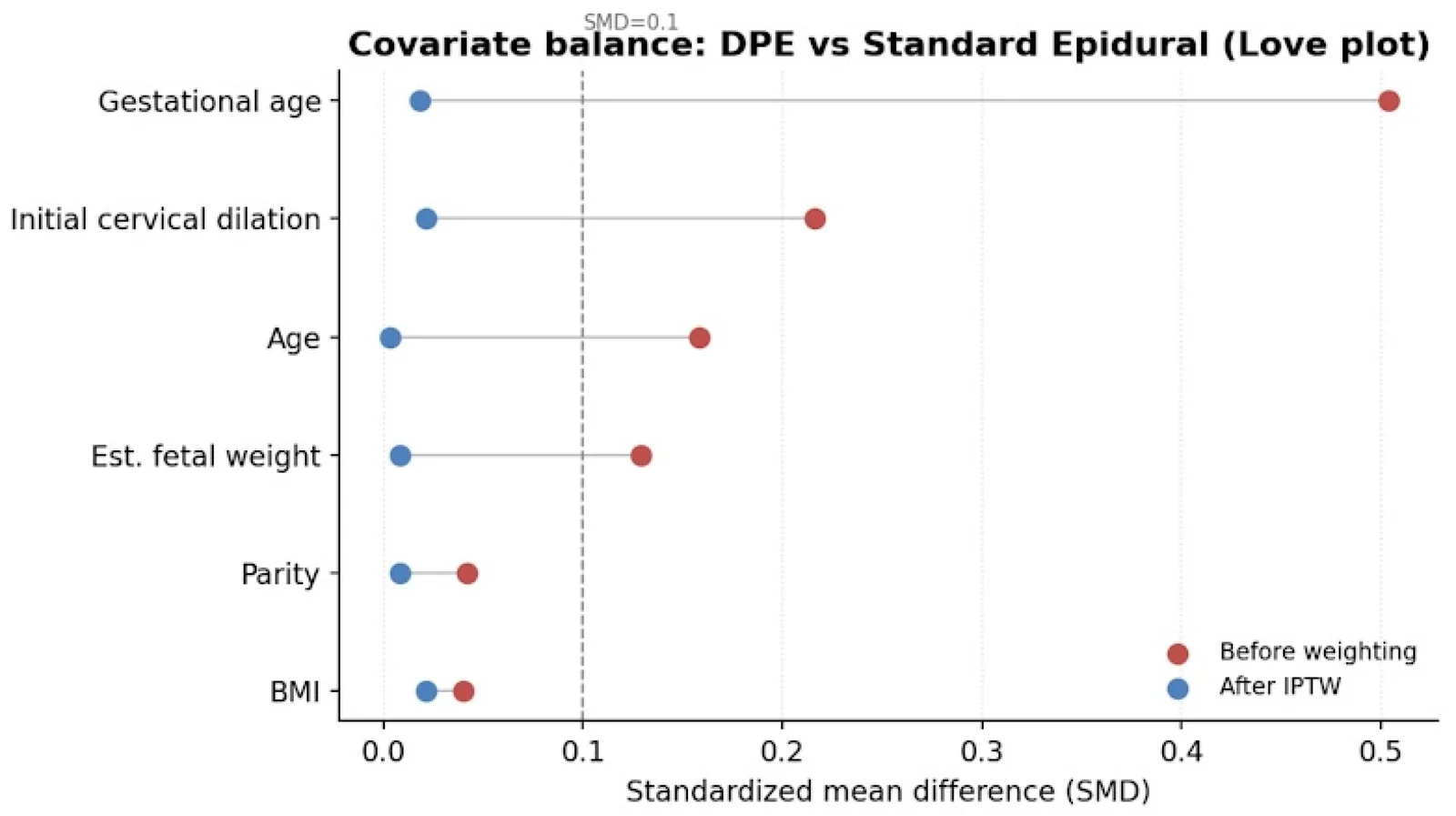
Covariate balance before and after propensity-score inverse probability of treatment weighting (Love plot), After weighing, all standardized mean differences (SMD) fall below 0.02, indicating good balance.

Propensity-score-based inverse probability of treatment weighting was specifically applied to compare the standard epidural and dural puncture epidural analgesia groups while improving balance in baseline covariates. After weighing, covariate balance improved markedly, and all standardized mean differences were reduced to below 0.05 (Figure 7).

In the weighted comparison, dural puncture epidural analgesia showed a small, non-significant absolute increase in biochemical acidosis compared with standard epidural analgesia, with a risk difference of approximately +2.5%. Weighted risk differences for recorded fetal acidosis (approximately +8.4%) and neonatal intensive care unit admission (approximately +5.1%) were likewise non-significant, although the direction of effect was preserved.

A sensitivity analysis restricted to nulliparous women (*n* = 89) was performed to further assess the influence of parity. Median second-stage duration was 60.5 min in the control group, 47.5 min in the standard epidural group, and 45.0 min in the dural puncture epidural group, with no statistically significant difference among groups (*p* = 0.672). Second-stage duration was numerically longest in the no-epidural control group and did not differ significantly between the two epidural techniques. Baseline characteristics within this nulliparous subgroup (*n* = 89) were not separately re-tabulated; given the limited subgroup size and non-randomized design, this finding is best interpreted as supportive evidence against epidural-related second-stage prolongation rather than as evidence that epidural analgesia shortened labor.

## 4. Discussion

In this retrospective cohort study, contemporary low-dose standard epidural and dural puncture epidural analgesia were not associated with prolongation of labor, active pushing time, or increased major obstetric intervention rates. The central clinical message is therefore reassuring: the epidural techniques evaluated in this cohort did not produce clinically meaningful adverse effects on labor progression. Compared with standard epidural analgesia, dural puncture epidural analgesia demonstrated numerically lower pain scores and a lower rescue bolus requirement, although these differences were not statistically significant. These findings suggest that dural puncture epidural analgesia may offer modest improvements in analgesic performance without compromising obstetric outcomes.

The labor-progression findings are particularly relevant because concerns about epidural-related prolongation continue to influence counseling and intrapartum decision-making. Contemporary reviews and propensity-score analyses emphasize that apparent epidural-related prolongation in observational datasets is often difficult to separate from parity, labor dystocia, timing of analgesia request, induction or augmentation, and institutional obstetric management [1,2,11]. Although second-stage duration was numerically highest in the dural puncture epidural group, this difference was not statistically significant, and quantile regression showed no prolongation at the median, 75th, or 90th percentiles, with all confidence intervals including zero. The adjusted mean model showed only a small dural-puncture-associated increase that was not consistent across analytic methods. The absence of prolongation at the upper percentiles is important because the clinical concern is not merely a small shift in median duration but the possibility of markedly prolonged labor.

Baseline imbalance explains why crude comparisons require cautious interpretation. The no-epidural group was older, more often multiparous, and had greater cervical dilation at initial assessment, whereas the epidural groups were predominantly nulliparous and had higher induction or augmentation rates. Because these factors strongly affect labor duration, adjusted analyses were essential to reduce confounding by indication, as also emphasized in modern review and propensity-score literature [1,2,11]. After adjustment, the crude differences were largely attenuated and, together with the nulliparous sensitivity analysis, in which the second stage was longest in the no-epidural group, did not provide consistent evidence of epidural-related labor prolongation.

Major obstetric intervention rates were also reassuring. Cesarean delivery rates were low and comparable across groups, instrumental vaginal delivery was rare, and the transfusion requirement was similar. No occiput posterior fetal head position was documented in the available records, which limits inference regarding fetal malposition, but the available obstetric outcomes do not suggest an increase in major intervention associated with either standard epidural or dural puncture epidural analgesia.

The comparison between standard epidural and dural puncture epidural analgesia showed a directionally favorable analgesic profile for dural puncture epidural analgesia. Pain scores were numerically lower, and rescue or top-up bolus requirement was reduced from 69.6% with standard epidural analgesia to 55.6% with dural puncture epidural analgesia, although this difference was not statistically significant (*p* = 0.183). This pattern is consistent with the proposed mechanism of dural puncture epidural analgesia, in which intentional dural puncture without intrathecal drug administration may facilitate limited transmeningeal spread of epidurally administered local anesthetic and opioid, improving sacral coverage and block quality. Recent meta-analytic evidence suggests that dural puncture epidural analgesia may modestly shorten analgesia onset and reduce unilateral or motor block, while randomized trials show that these advantages may be endpoint- and regimen-dependent [6,8,9]. Evidence from cesarean epidural-extension anesthesia also supports faster block onset after dural puncture epidural initiation, although that setting is distinct from maintenance labor analgesia [12].

These analgesia findings should be interpreted as hypothesis-generating rather than definitive. The study was powered for second-stage duration, not for detailed block-quality outcomes such as onset time, bilateral sacral blockade, asymmetric block, or maternal satisfaction. Previous randomized trials and meta-analyses have reported faster onset, improved sacral spread, fewer unilateral blocks, and lower top-up requirements with dural puncture epidural analgesia in some settings, although the magnitude of benefit appears to vary according to spinal needle gauge, maintenance regimen, local anesthetic dosing, and patient population [3,4,5,6,7,8,9,12]. Our findings are compatible with this literature but do not establish superiority.

The neonatal acid–base findings require careful framing. Objective umbilical cord venous pH did not differ significantly among groups, and biochemical acidosis (cord venous pH < 7.20) was rare and not significantly different between groups. The only neonatal endpoint that differed was a recorded fetal acidosis flag, which was more frequent in the epidural groups but was not corroborated by the objective cord-pH values, by lower 1 min or 5 min Apgar scores, by any need for neonatal resuscitation, or by a statistically significant increase in neonatal intensive care unit admission. Thus, the present data support a recorded-flag-versus-objective-measure distinction: a documentation-based acidosis label differed between groups, whereas objective acid–base measures and clinically relevant neonatal impairment did not. This interpretation is consistent with large cohort evidence showing no independent association between labor epidural analgesia and clinically important adverse neonatal outcomes, hypoxic–ischemic encephalopathy, or umbilical arterial pH < 7.20 after adjustment for confounding [10,13,14].

Several factors argue against interpreting the recorded fetal acidosis difference as direct evidence of neonatal harm. The epidural groups had higher induction or augmentation rates and were predominantly nulliparous, both of which may reflect longer or more intensively managed labor and may also influence documentation practices. Retrospective records may be affected by sampling timing, arterial versus venous source, specimen handling, and documentation quality, and the recorded fetal acidosis flag in particular is a documentation-based variable rather than a standardized laboratory threshold. We therefore consider it plausible that the between-group difference in this flag reflects, at least in part, documentation bias (more intensive labeling of acidosis in monitored, instrumented epidural labors), coding or charting variability across clinicians, or residual confounding by indication, rather than a true difference in fetal biochemical status; the dissociation from objective cord pH values in the same cohort is consistent with this interpretation, although a genuine but clinically limited biological effect cannot be excluded on the basis of these data alone. Because the objective acid–base analysis was based on umbilical venous rather than arterial pH, direct comparison with studies using arterial cord gases should be made cautiously. In addition, Firth penalized logistic regression generated wide confidence intervals that crossed the null value, indicating substantial imprecision rather than a stable estimate of risk. Large adjusted neonatal cohorts further support cautious interpretation of isolated acid–base or early Apgar differences when parallel clinical deterioration is absent [10,13,14]. Nevertheless, the recorded fetal acidosis difference should not be dismissed; it warrants prospective evaluation with standardized arterial and venous cord gas sampling, detailed oxytocin exposure data, maternal temperature monitoring, continuous hemodynamic recording, and fetal heart rate tracing analysis.

The main strength of this study is its clinically pragmatic three-group design, including no-epidural labor, standard epidural analgesia, and dural puncture epidural analgesia. A second strength is the use of complementary statistical approaches tailored to common problems in labor analgesia research. Multivariable adjustment addressed baseline imbalance, quantile regression tested for upper-percentile labor prolongation, Firth penalized logistic regression addressed rare neonatal events and separation, propensity-score weighting improved balance between epidural techniques, and nulliparous sensitivity analysis explored parity-related confounding. Together, these methods support the conclusion that the observed associations were not driven simply by unadjusted group differences.

These findings should also be interpreted in light of their generalizability. The study was conducted at a single tertiary center in Diyarbakır, Türkiye, with a specific institutional epidural protocol, patient population, and obstetric practice pattern; induction and augmentation rates, parity distribution, and baseline cervical dilation in our cohort may not reflect other settings. Centers with different neuraxial protocols, patient demographics, labor management practices, or case mix may observe different absolute rates of labor prolongation, obstetric intervention, or recorded fetal acidosis, even if the underlying direction of effect is similar. External validation in other populations and healthcare settings is therefore warranted before these findings are generalized beyond comparable single-center, contemporary low-dose epidural practice. Overall, the findings support the obstetric safety of contemporary low-dose epidural techniques in this cohort, while suggesting a possible analgesic advantage of dural puncture epidural analgesia. The recorded fetal acidosis signal should be presented cautiously and distinguished clearly from objective acid–base measures and clinical neonatal outcomes. Larger prospective studies are needed to confirm whether this documentation-based finding reflects residual confounding, measurement or coding variability, or a true but clinically limited effect.

### Limitations

This study has several limitations, which are summarized here. The retrospective, single-center design at a single Turkish tertiary center limits causal inference and generalizability to other populations and obstetric practice patterns; because group allocation was based on the analgesia technique actually received rather than randomization, selection bias and confounding by indication cannot be fully excluded, notwithstanding the multivariable adjustment, quantile regression, propensity-score weighting, and nulliparous sensitivity analysis used to address them. The study groups were also substantially imbalanced at baseline, with the no-epidural control group being older, more often multiparous, and further dilated at initial assessment, so residual and unmeasured confounding may still have influenced the results. Several clinically relevant variables were not available in sufficient detail because of the retrospective data source, including exact oxytocin dose and duration, detailed fetal heart rate tracing characteristics, maternal temperature trajectory, precise timing of epidural request relative to labor progression, standardized analgesia-onset timing, and gestational age, which was documented in only a subset of records and was therefore excluded from the adjusted models. Umbilical cord acid–base status was assessed from venous rather than paired arterial samples, which are less sensitive to acute intrapartum hypoxic events, limiting direct comparison with studies using arterial cord gases. The sample size was calculated for the primary outcome, second-stage duration, and the study may therefore have been underpowered to detect small but clinically meaningful differences in secondary outcomes, particularly between standard epidural and dural puncture epidural analgesia; several outcomes, including instrumental vaginal delivery, occiput posterior position, and neonatal resuscitation, were rare or absent in this cohort and could not be meaningfully evaluated. Finally, the study did not include detailed patient-reported outcomes such as maternal satisfaction, time to adequate analgesia, bilateral sacral blockade, or formal assessment of asymmetric block, so although dural puncture epidural analgesia was associated with a numerically lower, non-significant rescue bolus requirement, the study cannot fully characterize the comparative analgesic performance of the two epidural techniques.

In this retrospective cohort study, standard epidural and dural puncture epidural analgesia were not associated with clinically meaningful prolongation of labor or with increased major obstetric intervention, and dural puncture epidural analgesia showed a non-significant trend toward better analgesic performance. Objective neonatal acid–base measures did not differ between groups; the only difference was a recorded fetal acidosis flag that was not corroborated by cord pH or by adverse clinical neonatal outcomes. These findings support the obstetric and neonatal safety of contemporary low-dose epidural techniques, while underscoring the need to distinguish documentation-based markers from true neonatal impairment. Prospective studies with standardized cord blood gas sampling are warranted to clarify this isolated finding.

## Figures and Tables

**Table 1:** Baseline characteristics of the groups.

Variable	Control (*n* = 58)	Standard Epidural (*n* = 56)	DPE (*n* = 54)	*p*	Test
Age (years)	27.0 (22.0–31.8)	23.0 (21.0–26.0)	24.0 (21.0–27.0)	0.003	KW
BMI (kg/m^2^)	27.2 (24.5–29.6)	28.2 (26.0–29.9)	28.1 (25.5–30.7)	0.228	ANOVA
Parity	1 (0–3)	0 (0–1)	0 (0–1)	<0.001	KW
Gestational age (weeks)	39.0 (38.0–40.0)	38.7 (37.1–39.6)	39.3 (38.3–40.0)	0.027	KW
Initial cervical dilation (cm)	3 (2–5)	2 (2–3)	2 (1–3)	<0.001	KW
Estimated fetal weight (g)	3125 (2900–3400)	3000 (2788–3325)	3125 (2912–3300)	0.375	ANOVA
Induction/augmentation, *n* (%)	46 (79.3)	55 (98.2)	51 (94.4)	0.001	χ^2^

Continuous variables are median (IQR) for Kruskal–Wallis (KW) and mean ± SD for ANOVA. BMI: body mass index.

**Table 2:** Comparison of the course of labor and obstetric outcomes among groups.

Variable	Control (*n* = 58)	Standard Epidural (*n* = 56)	DPE (*n* = 54)	*p*	Test
First-stage duration (min)	195 (120–300)	162 (90–280)	180 (101–275)	0.619	KW
Second-stage duration (min)	40.0 (26.2–61.2)	45.0 (35.0–60.0)	48.5 (37.2–60.0)	0.118	KW
Active pushing (min)	8.0 (4.0–21.0)	8.5 (3.0–16.0)	12.0 (5.2–26.8)	0.292	KW
Blood loss (mL)	450 (400–508)	540 (360–720)	540 (360–720)	0.091	KW
Cesarean delivery, *n* (%)	1 (1.7)	2 (3.6)	1 (1.9)	0.773	Fisher
Instrumental vaginal delivery, *n* (%)	1 (1.7)	0 (0.0)	0 (0.0)	0.385	Fisher
Occiput posterior position, *n* (%)	0 (0.0)	0 (0.0)	0 (0.0)	1	–
Blood transfusion, *n* (%)	2 (3.4)	2 (3.6)	1 (1.9)	0.84	Fisher

Continuous variables are presented as median (IQR) and compared with the Kruskal–Wallis (KW) test; categorical variables are presented as *n* (%) and compared with Fisher’s exact test. IQR: interquartile range; DPE: dural puncture epidural.

**Table 3:** Comparison of neonatal outcomes among groups.

Variable	Control (*n* = 58)	Standard Epidural (*n* = 56)	DPE (*n* = 54)	*p*	Test
APGAR 1 min	8 (8–8)	8 (8–8)	8 (8–8)	0.416	KW
APGAR 5 min	9 (9–9)	9 (9–9)	9 (9–9)	0.756	KW
Umbilical cord pH	7.380 (7.360–7.400)	7.367 (7.335–7.395)	7.370 (7.345–7.401)	0.266	KW
Birth weight (g)	3150 (2802–3490)	2980 (2750–3270)	3135 (2872–3368)	0.389	ANOVA
Acidosis (cord pH < 7.20), *n* (%)	0 (0.0)	1 (1.8)	2 (3.7)	0.335	Fisher
Fetal acidosis (recorded), *n* (%)	0 (0.0)	4 (7.1)	9 (16.7)	0.004	Fisher
NICU admission, *n* (%)	3 (5.2)	6 (10.7)	9 (16.7)	0.145	χ^2^
Neonatal resuscitation, *n* (%)	0 (0.0)	0 (0.0)	0 (0.0)	1	–
Phototherapy, *n* (%)	3 (5.2)	0 (0.0)	0 (0.0)	0.055	Fisher

NICU: neonatal intensive care unit.

**Table 4:** Adjusted odds ratios for biochemical acidosis from Firth penalized logistic regression.

Variable (Outcome: Cord pH < 7.20)	Adjusted OR	95% CI	*p*
DPE (vs. Control)	5.57	0.25–122	0.275
Standard Epidural (vs. Control)	3.16	0.12–82	0.487

Adjusted odds ratios (OR) for biochemical acidosis (cord pH < 7.20) from Firth penalized logistic regression (group-only model; only 3 events). CI: confidence interval.

## Data Availability

The data presented in this study are available from the corresponding author upon reasonable request. The data are not publicly available because they contain information that could potentially compromise participant privacy and are subject to institutional ethical restrictions.

## Supplementary Materials

The following supporting information can be downloaded at https://www.mdpi.com/article/10.3390/jcm15166380/s1, STROBE Statement—Completed Checklist.
