# Supplementary material for: Dural Puncture Epidural, Standard Epidural, and No Epidural: Comparative Effects on Labor Progression and Neonatal Outcomes
Source: J Clin Med. 2026 Aug 18;15(16):6380. doi: 10.3390/jcm15166380 (PMC13513571; doi:10.3390/jcm15166380)
Supplement: Supplementary file 1 [file jcm-15-06380-s001.zip › jcm-4471449-supplementary.pdf]

STROBE Statement – Completed Checklist

Item	Recommendation	Page No.	Relevant text from manuscript
1a	Indicate study design in title/abstract	1–2	Retrospective observational cohort study identified in abstract.
1b	Provide informative abstract	2	Structured abstract (Objective, Methods, Results, Conclusion).
2	Background/rationale	2–4	Scientific background and rationale.
3	Objectives	4	Primary and secondary objectives with hypothesis.
4	Study design	4	Single-center retrospective observational cohort study.
5	Setting	4	Hospital setting, study dates, ethics approval, data sources.
6a	Participants	4–5	Eligibility criteria, participant selection, grouping.
6b	Matching	N/A	Not applicable. Unmatched retrospective cohort study.
7	Variables	7–8	Definitions of outcomes, exposures, confounders, diagnostic criteria.
8	Data sources/measurement	4–8	Hospital, anesthesia, obstetric and neonatal records.
9	Bias	8–9, 11–15	Adjusted analyses, propensity weighting, Firth regression, sensitivity analyses.
10	Study size	8	Sample-size calculation and achieved enrollment.
11	Quantitative variables	7–9	Handling of continuous and categorical variables.
12a	Statistical methods	8–9	Complete statistical methods.
12b	Subgroups	8–9,13	Nulliparous subgroup and sensitivity analyses.
12c	Missing data	7–9,15	Gestational age missing; excluded from adjusted models.
12d	Follow-up/matching	N/A	Not applicable. No follow-up, matching, or complex sampling.
12e	Sensitivity analyses	9,13	Sensitivity and weighted analyses.
13a	Participants	5,9,21	Participant numbers reported; flow diagram.
13b	Non-participation	5,21	Reasons for exclusions.
13c	Flow diagram	21	Figure 1.
14a	Descriptive data	9,17	Baseline characteristics (Table 1).
14b	Missing data	7–9,15	Missing gestational-age data described.
14c	Follow-up time	N/A	Not applicable. Outcomes assessed during index hospitalization.
15	Outcome data	9–13,18–20	Labor, maternal, analgesic, and neonatal outcomes.
16a	Main results	9–13	Adjusted and unadjusted estimates reported.
16b	Category boundaries	7–8	Definitions of categorized variables.
16c	Absolute risk	N/A	Not applicable. Absolute frequencies and percentages reported.
17	Other analyses	10–13	Quantile regression, IPTW, Firth regression.
18	Key results	13–15	Summary of principal findings.
19	Limitations	16–17	Study limitations discussed.
20	Interpretation	13–17	Balanced interpretation.
21	Generalisability	16–17	External validity discussed.
22	Funding	17	No external funding.
